# A Late Presentation of Substance-related Rhabdomyolysis with Normal Serum Creatine Kinase Levels and Complicated with Acute Tubular Necrosis

**DOI:** 10.7759/cureus.4197

**Published:** 2019-03-06

**Authors:** Asrar Ahmad, Muhammad A Zain, Ammar A Ashfaq, Waqas Ullah

**Affiliations:** 1 Internal Medicine, Abington Hospital-Jefferson Health, Abington, USA; 2 Internal Medicine, Sheikh Zayed Medical College and Hospital, Rahim Yar Khan, PAK

**Keywords:** rhabdomyolysis, rhabdomyolysis-induced renal failure, acute tubular necrosis, myoglobin induced acute kidney injury, substance abuse related acute tubular necrosis, heroine induced acute tubular necrosis, delayed presentation of rhabdomyolysis

## Abstract

Substance abusers are at increased risk of acute kidney injury (AKI) compared to the general population due to nontraumatic rhabdomyolysis. The primary target of these nephrotoxic agents is the tubulointerstitial compartment and the most frequent findings on biopsy are consistent with acute tubular necrosis (ATN) and acute interstitial nephritis. We present a rare case of an intravenous cocaine and heroin abuser who presented with recent onset oliguria, hematuria, and reduced peroral intake. The urine dipstick testing showed guaiac positivity but no red blood cells on microscopy. The blood workup showed elevated serum creatinine and urea levels but normal creatinine kinase (CK) level. Renal biopsy showed tubular epithelial cell necrosis and positive immunoperoxidase staining for myoglobin pigment casts in renal tubules. The diagnosis of rhabdomyolysis-associated ATN secondary to substance abuse was suggested. However, normal serum CK levels as well as urine drug panel supported the delayed presentation of rhabdomyolysis complicated with ATN. The patient returned to normal health with no residual kidney dysfunction after undergoing temporary hemofiltration.

## Introduction

Heme pigment (HP)-containing protein molecules such as myoglobin and hemoglobin are essential for the normal functioning of myocytes and red blood cells (RBC), respectively. These HP-containing proteins are spilled into the bloodstream if an injury occurs to myocytes (rhabdomyolysis) or red blood cells (hemolysis). Rhabdomyolysis (RM), regardless of the underlying cause, stems from necrosis of myocytes and results in spillage of myocyte contents into the bloodstream. These include electrolytes and myoglobin and muscle enzymes that include lactate dehydrogenase (LDH), creatinine kinase (CK), aldolase, aspartate aminotransferases (AST), and alanine aminotransferases (ALT) [1]. RM may present as an incidentally raised serum CK level, myalgias, myoglobinemia, myoglobinuria, acute tubular necrosis (ATN) or life-threatening presentations such as electrolyte imbalance and cardiopulmonary complications.

Major trauma-related crush injuries are the most common cause of RM, but recreational substance abuse, such as heroin and cocaine abuse, has also been established to cause RM [2]. Classically, RM-associated ATN following substance abuse presents with many fold rise in plasma CK levels and myoglobinuria [3], but we are reporting a unique presentation of RM-associated ATN with normal serum CK levels and positive immunoperoxidase staining for myoglobin tubular casts on renal biopsy. The patient acknowledged the recent substance abuse about more than a week ago. However, a urine drug screen testing showed a negative result. The higher creatinine levels upon presentation with normal serum CK titers and positive myoglobin staining renal tubular casts suggested the delayed presentation of substance-related RM complicated with ATN until the normalization of serum CK levels.

RM comprises about 7%-10% of cases of AKI in the form of ATN, while approximately 13%-50% of cases of RM, if not diagnosed and treated earlier, go on to develop AKI [4,5]. So the early diagnosis of RM can prevent the progression to AKI. Currently, serum CK levels are used as a screening test for the diagnosis of RM but since CK levels return to baseline after 6-10 days on average [6], fewer undiagnosed or missed RM cases may go on to develop ATN. The cases, like ours, seeking delayed medical attention with established ATN and normalization of serum CK levels may be difficult to diagnose. Our study reports the successful use of invasive renal biopsy testing and immunoperoxidase staining for detecting myoglobin renal tubular casts to differentiate the underlying cause of AKI. The purpose of this case is to make the physician aware of an unusual presentation of muscle injury following intravenous drug abuse (heroin and cocaine) and highlight the need for an alternative non-invasive diagnostic tool for the diagnosis of RM-associated ATN with normal serum CK levels.

## Case presentation

A 36-year-old male intravenous drug abuser of cocaine and heroin with a history of chronic hepatitis C infection for the last 12 years presented to the emergency room (ER) with complaints of nausea, abdominal pain, decreased urine output, and dark discoloration of his urine for three days. He had reduced peroral intake due to nausea, and he otherwise denied having a recent trauma, excessive exercise, medications intake, fevers, chills, shortness of breath, dark discoloration or pale stools. He denied any current recreational drug use, but the family feared that his recent behavior over a week ago suggested recent substance abuse. His vitals upon presentation were as follows: he was afebrile and he had a blood pressure of 120/80 mmHG and a heart rate of 70 beats per minute (bpm).

Investigation

**Figure 1:**
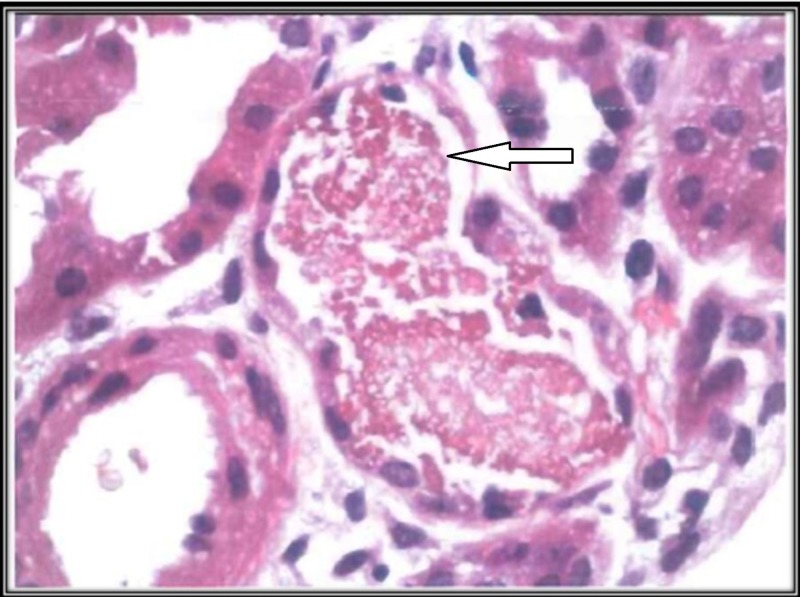
Renal biopsy showing positive immunoperoxidase staining for myoglobin pigmented cast in renal tubular lumen (white arrow).

His blood work done in the ER showed elevated creatinine (Cr) of 8.26 milligrams per deciliter (mg/dl) and a blood urea nitrogen (BUN) level of 55 mg/dL. His serum sodium was 130 milliequivalents per litre (mEq/L), potassium was 5.2 mEq/L, calcium was 4.7 mEq/L, and serum CK without reflexive MB were 152 units per liter (U/L). His serum lactate dehydrogenase (LDH) was 210 U/L, serum ALT 114 U/L, and AST 54 U/L. His hemoglobin (Hb) level was 14.7 gram per deciliter (g/dL) with a normal reticulocyte count, peripheral blood smear (PBS), clotting profile and negative Coombs testing. His urine dipstick showed gross hematuria, specific gravity of 1.03 and 3+ proteinuria. A urine microscopy test showed normal 1-3 red blood cells (RBC) per high power field (HPF). A urine toxicology screen was negative. Computed tomography of the abdomen and pelvis revealed no evidence of a stone. A serum assay for complement was within standard limits. A serum panel for antinuclear antibodies (ANA), antinuclear cytoplasmic antibodies (ANCA) and anti-glomerular basement membrane antibodies (anti-GBM) levels came back negative. Arterial blood gas (ABG) showed pH of 7.34, pCO2 of 43, HCO3 of 24. After an extensive workup, our suspicion was cryoglobulinemia-induced rapidly progressive glomerulonephritis (RPGN) secondary to the long-standing history of hepatitis C virus (HCV) infection. A left-sided kidney biopsy was consistent with normal glomerular histology on gross and electron microscopy, and pigmented tubular casts (Figure 1). The immunoperoxidase staining was positive for myoglobin pigmented cast in distal renal tubules. The biopsy findings were consistent with HP-induced ATN or RM-induced ATN secondary to substance-induced RM.

Differential diagnosis

On presentation, we suspected cryoglobulinemia-induced RPGN secondary to the long-standing history of HCV infection. We were also suspicious that it could be hemoglobinuria-associated AKI due to the findings of heme positivity on urine dipstick with negative urine microscopy for RBC, but a normal PBS, reticulocyte, and bilirubin were not consistent with this etiology.

Treatment

He did not have any indication for urgent dialysis. Due to persistently abnormal kidney function with oliguria and kidney biopsy pointing towards RM-induced ATN, the patient was placed on hemofiltration (continuous veno-venous hemofiltration/CVVH) after placing a temporary catheter. The abnormal liver function test was suggested to be due to RM according to hepatology consult. So no specific action was advised regarding it.

Outcome and follow-up

His urine output started improving on Day 5 with supportive therapy. His Cr dropped significantly after undergoing temporary hemofiltration. On the 10th day of admission, his serum Cr was 1.3 and BUN was 25. He was discharged home with a close follow-up with nephrology. On follow-up at the one-month interval, his renal function was within standard limits with a Cr of 0.9 and BUN of 15. His liver enzymes had also returned to normal values at the one-month follow-up visit.

## Discussion

Approximately 26,000 cases of RM are reported annually in the United States [7]. The potential causes of RM are broadly categorized as traumatic (e.g., crush injuries), nontraumatic exertional (e.g., marked physical exertion in a previously untrained individual), and nontraumatic non-exertional (e.g., metabolic myopathies, electrolyte disorders, seizure disorders, hyperthermia, prolonged immobilization, drugs or toxins) [1]. Nontraumatic non-exertional RM, such as in our patient, can be rarely triggered by abuse of recreational drugs such as heroin or cocaine. The underlying pathology in RM, irrespective of the underlying cause, is myocytes necrosis. Heroin causes prolonged sustained posture or immobilization, which leads to muscle ischemia due to compression of muscle and capillaries, while cocaine may cause severe arterial vasoconstriction of vessels supplying skeletal muscles by inhibition of catecholamine reuptake at the alpha adrenergic receptors in muscles and end up causing increased intracellular free ionized calcium [8, 9].

Regardless of the underlying cause of RM, the final common pathway in muscle necrosis is either activation of the proteases enzymes involved in cell death due to elevated cytoplasmic and mitochondrial free ionized calcium or cell membrane lysis due to ischemia-induced decreased ATP production [9]. Myocytes damage results in spillage of cellular contents, such as muscle enzymes (LDH, aspartate aminotransferases, alanine aminotransferases, aldolase, and CK), electrolytes and myoglobin into the bloodstream [8]. Among those, the culprit agent for clinical presentation and pathological consequences of RM is myoglobin [9]. Once released into the bloodstream, myoglobin is filtered into the urinary space where it can result in the formation of pigmented casts in the renal tubular lumen. These pigmented casts compromise glomerular filtration by blocking the tubules. Other possible mechanisms of ATN are the direct toxic effect of HP on the tubular epithelial cell and HP-induced vasospasm of the artery supplying the renal medulla [10, 11]. Despite the toxic effects on the renal tubulointerstitial interface, myoglobin rarely causes AKI unless other predisposing factors are present concomitantly such as volume depletion, ischemia or metabolic acidosis [11]. In our case, it was also suspected that the patient was not able to maintain his hydration status while being under the effect of cocaine and heroin, which might have triggered the progression of RM to AKI.

Cocaine abusers might incidentally be diagnosed with RM based on their higher serum CK level while presenting with other complaints related to cocaine abuse such as fever, seizure, cardiac arrhythmias, or chest pain. However, few cases might present with myalgias or ATN itself. Merigian et al. first described the association between cocaine abuse and RM-associated ATN in 1987 [12]. Recreational drugs abuse may cause RM and ATN with any dose or route of drug administration.

Although the pathogenesis of RM-associated ATN may correlate more closely with plasma myoglobin levels, it is seldom used in making a diagnosis because it returns to normal serum values within six to eight hours or at the time of presentation, due to a shorter half-life of two to three hours and quicker metabolism by the liver. Because serum CK has a longer half-life of about 36 hours, a five-fold rise in serum CK levels from baseline is diagnostic for RM [5]. Serum CK levels are also used in RM cases to predict the risk of progression to AKI. The serum CK value of >15000 U/L has been shown to correlate strongly for the development of AKI in a patient with RM [13]. Based on normal serum CK levels, we overlooked the possibility of RM to be the underlying cause of AKI. We did not measure the myoglobin levels of our patient. So in patients, like our case, who have RM-related ATN with normal serum CK levels, the serum CK and myoglobin levels are not reliable tools for diagnosing the underlying cause of ATN. Since the presence of hematuria on dipstick testing and absence or 1-2 normal RBC on urine microscopy is consistent with both hemolysis and RM, it is not a sensitive test for the diagnosis of RM. However, hemolysis can be ruled out by a normal Hb, reticulocyte count, bilirubin, and PBS. In RM-associated ATN patients, urine dipstick testing might show some mild or moderate level of proteinuria too [4]. The lower BUN to CR ratio is also expected in RM-associated ATN due to a rapid rise in serum creatinine levels, which is suggestive of the intrarenal cause of AKI [14]. Although hypotension, the most common cause of prerenal azotemia, can also cause ATN with the presence of granular cast on urine microscopy. However, the absent urinary granular casts and positive staining for myoglobin pigmented casts on renal biopsy inferred that any recent hypotension did not result in the ATN.

Treatment of RM is targeted at the underlying cause and prevention of AKI development by maintaining a good hydration status and diuresis, which increases clearance of myoglobin from the body. If patients with RM of any reason present with increasing serum CK levels regardless of baseline serum CK levels, then they should be started on intravenous fluids to prevent the progression to AKI. The fluid of choice is isotonic saline at the rate of 1-2 liter per hour. Diuretics such as loops are only given if the patient develops pulmonary edema secondary to excessive fluid resuscitation. The use of bicarbonates and mannitol to clear myoglobin through kidneys has also been proposed to prevent progression of RM to AKI. However, this is not supported by the data provided by studies [15].

In patients with established AKI, like ours, who have oliguric RM-associated ATN, maintenance of fluid and electrolyte balance is the mainstay of treatment, and temporary high flow dialysis can also be tried. The fluids should be given with caution as they can deteriorate into lethal cardiopulmonary complications of electrolyte derangements and pulmonary edema secondary to fluid overload. Myoglobin levels return to normal within 24 hours of injury, due to shorter half-life, which is why it is suggested to be cleared from serum in an oliguric patient, such as ours, to halt the ongoing kidney injury. Myoglobin has a minimal molecular weight of approximately 18 kD, so it can be best cleared with highly porous and high cut off ultrafiltration membrane filters in continuous venovenous hemofiltration (CVVH) than conventional dialysis [16]. However, satisfying data is not available as to whether better myoglobin clearance affects the recovery of renal function in patients with RM. Metabolic abnormalities, which include hyperkalemia, hypocalcemia or hypercalcemia, hyponatremia, hyperuricemia, hypomagnesemia, metabolic acidosis, and hyperphosphatemia, are relatively common in patients with RM-associated AKI. That is why serum electrolytes should be monitored on a daily basis until they become normal. Calcium supplements should be given in case of symptomatic hypocalcemia or hyperkalemia. Patients with RM-associated AKI have a favorable prognosis overall with an approximate mortality rate of 20% [16].

## Conclusions

Acute kidney injury (AKI) should be recognized as a possible complication of rhabdomyolysis (RM) secondary to substance abuse (heroin and cocaine abuse). The most common presentation of substance-related rhabdomyolysis is elevated serum creatine kinase levels but cases with missed episodes of substance-related RM or late presentation of RM might report established AKI with normal serum CK. Thus serum CK levels are not a reliable diagnostic tool for such cases. We report the successful use of immunoperoxidase staining on renal biopsy samples for myoglobin pigmented casts in renal tubular lumens for identifying the underlying cause of AKI. It is thus recommended that a noninvasive screening test should be identified to better diagnose such cases.
